# 

**Published:** 1852-07

**Authors:** 


### CONTENTS

### OF NO. I.

### ORIGINAL COMMUNICATIONS.

### ESSAYS AND CASES.

Art. I.—Water as a Remedy in Disease. By John C. Darby, M. D., of Lexington, Ky., - - - - - - 1
Art. II.—On the Treatment of Dysentery. By Robert Durrett, M. D., Resident Graduate at the Louisville Marine Hospital, during the year 1851. - - - - - - - 17
Art. III.—A Monograph on Fractures: Being an Inaugural Dissertation submitted to the Trustees and Medical Faculty of the University of Louisville, March, 1852. By James Knapp, M. D., of Louisville, Ky. - - - - - 18
Art. IV.—Chemical Examination of the Mineral Water of Cooper's Well, near Raymond, Mississippi. By J. Lawrence Smith, M. D., Professor of Chemistry in the University of Louisiana. - - - - - - - - 30

### BIBLIOGRAPHICAL NOTICES.

Art. V.—Tableaux of New Orleans. By Bennet Dowler, M.D. 34
Art. VI.—The East Tennessee Record of Medicine and Surgery. Edited by Frank A. Ramsey, A. M., M. D. - 48

### SELECTIONS FROM FOREIGN AND HOME JOURNALS.

Cases treated with Veratrum Viride, - - - - - - - 49
Veratrum Viride, as a Poison, - - - - - - - - - 56
The Itch Cured in Two Hours, - - - - - - - - 58
Treatment of Obstinate Ulcers by the Internal Use of Tincture of Cantharides, - - - - - - - - - - 59
On the Use of Full Doses of Opium in Continued Fevers, - 60

Anesthesia, - - - - - - - - - - - 62
On the Action of Ergot of Rye, &c. - - - - - - - 65
Contagion of Typhoid Fever, - - - - - - - - 68
Pathology of Asthma, - - - - - - - - - - 71
American Medical Association, - - - - - - - - 74
On the Distribution of the Crinoidea in the Western States, - 79
Quinine in Typhus and Typhoid Fevers, - - - - - 86

ORIGINAL INTELLIGENCE.

Contagiousness of Typhoid Fever, - - - - - - - 88
The Spiritual Rappers Once More, - - - - - - 89
Association of Botanists, - - - - - - - - - 90
History of Surgery in Kentucky, - - - - - - - 91
University of Louisville, - - - - - - - - 91
University of Nashville, - - - - - - - - 92

